# Dual Effect of Ziconotide in Primary Erythromelalgia

**DOI:** 10.1155/2015/592170

**Published:** 2015-11-02

**Authors:** Rosario Russo, Maria Cristina Caroleo, Erika Cione, Mariarita Perri, Maria Teresa Paparo, Antonio Russo

**Affiliations:** ^1^Pain Therapy Unit, Department of Hematology and Oncology, Pugliese-Ciaccio Hospital, Catanzaro, Italy; ^2^Department of Pharmacy and Health and Nutritional Sciences, University of Calabria, Italy; ^3^Molecular Biology Unit, Department of Hematology and Oncology, Pugliese-Ciaccio Hospital, Catanzaro, Italy

## Abstract

Erythromelalgia (EM) is a rare disabling clinical syndrome more commonly known to affect the lower extremities. There is no single effective treatment for this disease that often requires a multidisciplinary approach. Herein, we report the case of a 31-year-old woman affected by primary erythromelalgia who was successfully treated with intrathecal Ziconotide. We also observed an unexpected result following therapy with Ziconotide. The legs and feet of the patient that at the time of admission were swollen and tumefied dramatically improved after one week of the drug administration.

## 1. Introduction

EM is a rare clinical syndrome characterized by intermittent heat, redness, swelling, and pain more commonly affecting the lower extremities [1]. Symptoms are mostly aggravated by warmth and are eased by a cold temperature [2]. In some cases, symptoms can be very severe and disabling. Erythromelalgia can be classified as either familial or sporadic, with the familial form inherited in an autosomal dominant manner [3]. There is no single effective treatment for erythromelalgia and therapeutic management of this disease is very difficult and there is a need of a multidisciplinary approach.

Herein, we report the case of a 31-year-old woman affected by primary erythromelalgia that after several ineffective treatments was successfully treated with intrathecal Ziconotide. Furthermore, we have described an unexpected effect of this drug.

## 2. Case Presentation

**Figure 1:**
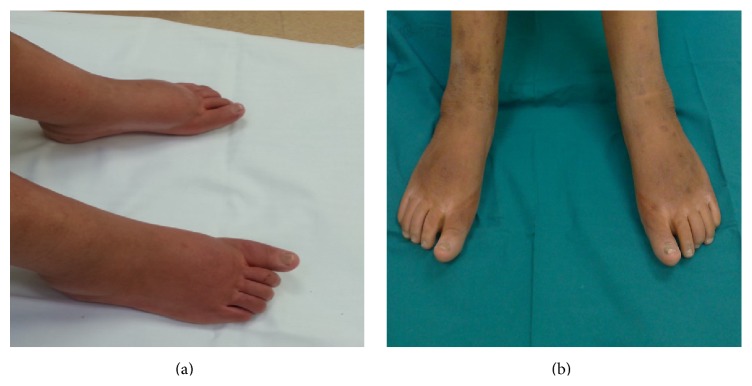
Patient legs and feet at the time of admission (a) and after one week of Ziconotide treatment (b).

**Figure 2:**
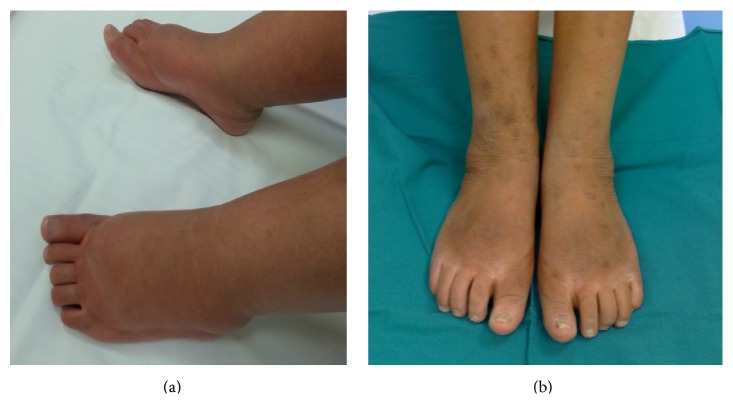
Patient legs and feet delaying pump recharging of 4 days (a) and after one week of pump recharging with usual dosage of Ziconotide (b).

The patient, a 31-year-old Caucasian female, was diagnosed with primary EM at the age of 18. She was later diagnosed with exophthalmos and concomitant megalocornea and with bilateral congenital glaucoma resulting in loss and severe impairment of vision at the level of right and left eye, respectively. The symptoms of primary erythromelalgia started at the age of 6 with intermittent painful skin redness and swelling of both her feet and lower parts of the legs. Over time her symptoms increased in frequency and severity often requiring hospitalization. A chronic treatment with NSAIDs and exposure of lower legs to cold compresses were provided resulting in an improvement of symptoms. She was hospitalized once more in October 2008, because the worsening of symptoms evolved into constant erythema and warmth of the lower extremities with associated pain. Her symptoms were aggravated even further by exertion, stress, weight-bearing, and gravity dependency and she spent most time immersing her feet in cold water. She could not wear socks or shoes or cover her feet or legs and she was unresponsive to any of her current medications. Because of severe pain with intense burning to the extremities, the patient was then moved from the Dermatology Unit to our division. Physical examination revealed increased skin temperature to the lower extremities, very strong burning pain, and swollen ankles. There was evidence of chronic immersion injury to the skin on her feet which was thickened, reddened, and macerated with ulcerations. The patient rated her pain as 10/10 on a 0 to 10 scale value (numeric rating scale pain intensity, NRSPI) when lying completely still. She displayed secondary allodynia at the level of the perimalleolar areas and bilateral hyperalgesia at the level of the gastrocnemius and of the instep. As first line of treatment, we started with pregabalin 75 mg twice a day and oxycodone 5 mg twice a day. The drug dosage was then increased in the following weeks up to 150 mg twice a day and 20 mg twice a day for pregabalin and oxycodone, respectively. This therapeutic regimen yielded a considerable improvement of the symptoms such as pain relief (NRSPI score: 3-4), decreased burning sensation to the extremities with need for less cold medications, and regular nocturnal sleep; however, the swelling of lower limbs remained unchanged. The improvement of symptoms lasted until February 2010. In March 2010, the patient was again hospitalized in our unit, since she reported an escalation of pain without apparent triggers. The disabling pain was unresponsive to the therapy; thus, we decided to perform opioid rotation and to replace the anticonvulsant. However, the increase of the drugs yielded heavy side-effects as drowsiness, constipation, and profound asthenia, so we decided in favor of an implantable intrathecal pump drug delivery system. To determine whether the patient will benefit from an implant we performed a proper trial phase. During the trial, the planned drugs were infused through an indwelling catheter that was placed intrathecally. We started with the administration of an anaesthetic at low dose followed by administration of an opioid at low dose 24 hours later. The trial performed with administration of 0.03 mg morphine revealed an improvement of painful symptoms; however, the appearance of intense itching on her face occurred; thus, we decided to use Ziconotide. A fortnight after the test the patient underwent the implant of a spinal port a cache for Ziconotide titration in our unit. The drug was administered through a micropump (CADD 7300 MS3-7400 model) following a low titration schedule starting from the dosage of 0.3 mcg/*die* to the dosage of 1.2 mcg/*die*. As a result we observed a clear-cut improvement of the symptoms with pain relief (NRSPI score: 3) and disappearance of allodynia and hyperalgesia. The administration of the anticonvulsant was interrupted and the dosage of opioid, orally administered, was reduced to 5 mg twice a day. At the end of titration the patient was again hospitalized once more in our division to be subjected to the final implant of the intrathecal pump drug delivery system (10 mL Tricumed model/delivering 0.26 mL/daily) with a dosage of 1.8 mcg/*die* of Ziconotide. We also observed an unexpected result following administration of Ziconotide: the legs and feet of the patient which at the time of admission were swollen and tumefied dramatically improved after one week (Figure 1). In April 2013, the patient made the pump recharging with a delay of 4 days and came to our observation with her legs and feet swollen along with burning pain. Two days after refill with the usual dosage of Ziconotide the legs were no longer swollen, with no burning pain and with noticeable improvement after one week (Figure 2).

## 3. Discussion

A universally effective treatment for EM is unknown. The mainstay of therapy is support and avoidance of trigger factors. Aspirin is more effective in patients with secondary EM caused by myeloproliferative disorders [4, 5]. Sodium nitroprusside may be helpful in children [6]. Neuroactive drugs, including SSRIs, tricyclic antidepressants, gabapentin, pregabalin, and benzodiazepines, have had some symptomatic benefits in a few cases [4, 5] similar to surgical sympathectomy [4, 7]. NSAIDs and other types of analgesics and narcotics administered by different routes can be carefully used for pain control [4]. In this context it is worth mentioning that many patients with severe chronic pain fail to receive satisfactory pain relief with systemic or intrathecal (IT) opioid therapy [8–10]. Indeed, chronic pain is often either refractory or minimally responsive to long-term opioid treatment [8]. Furthermore, there are various limitations to the effectiveness of opioids in this population, including the risk of addiction and abuse [11, 12], the potential for loss of efficacy due to the development of tolerance [8, 12], and adverse events [12, 13]. The nonopioid analgesic Ziconotide has been developed as a new treatment for patients with severe chronic pain who are intolerant of and/or refractory to other analgesic therapies. Ziconotide is the synthetic equivalent of a 25-amino-acid polybasic peptide found in the venom of the marine snail* Conus magus* [14]. The mechanism of action of Ziconotide involves a potent and selective block of neuronal N-type voltage-sensitive calcium channels at the presynaptic level [15, 16], thereby inhibiting neurotransmission from primary nociceptive afferents. Ziconotide produces potent antinociceptive effects in animal models [17] and its efficacy has been demonstrated in human studies [18]. The analgesic efficacy of Ziconotide likely results from its ability to interrupt pain signaling at the level of the spinal cord. Importantly, prolonged administration of Ziconotide does not lead to the development of addiction or tolerance [19].

In this report, we described the case of woman diagnosed with primary EM that after several ineffective treatments was successfully treated with intrathecal Ziconotide. The drug has significantly reduced the burning pain at lower extremities resulting in an improved quality of life of the patient allowing her to rest in the bed and not up in a chair for months. In addition, Ziconotide has eliminated the need for the patient to keep her feet in cold water, allowing an improvement of skin lesions caused by chronic immersion. We also observed an unexpected result following administration of Ziconotide: the legs and feet of the patient that at the time of admission were swollen and tumefied dramatically improved after one week. The mechanism through which Ziconotide exerts this effect is unknown. We hypothesized that, in our patient, EM is associated with a concomitant process of neuroinflammation as described for several chronic pain conditions [20]. Neuroinflammation depends upon the release of inflammatory neuromodulators from nociceptive primary afferent nerve terminals. In particular, the current accepted model of this process is based on the capability of stimulating events to activate peripheral nociceptive nerve endings leading to the generation of impulses which are conducted antidromically through axon reflexes and orthodromically to the spinal cord [21, 22]. This latter event causes primary afferent depolarization that initiates dorsal root reflexes, which send additional antidromic impulses to the periphery [23]. As a result, neuropeptides are released from the peripheral nerve terminal and initiate neuroinflammation. In particular, the secretion of substance P, neurokinin A, and CGRP from activated C-fibre terminals produces intense protein plasma extravasation (oedema) and vasodilation [24, 25]. In this frame Ziconotide could interfere with the neuroinflammatory circuit leading to inhibition of the vasoactive response, as this drug through its selective and potent action on N-type voltage-sensitive calcium channels is able to control neurotransmission at many synapses [19].

Collectively, these results indicate that Ziconotide could be a novel drug in the management of chronic pain in EM. Furthermore, the unexpected improvement of leg and feet swelling and oedema after the treatment with the drug is a matter that deserves more attention for the use of Ziconotide also in other vasculopathies.
